# Extensive myelitis associated with anti-NMDA receptor antibodies

**DOI:** 10.1186/1471-2377-13-211

**Published:** 2013-12-27

**Authors:** Olivier Outteryck, Guillaume Baille, Jérôme Hodel, Marianne Giroux, Arnaud Lacour, Jérôme Honnorat, Hélène Zéphir, Patrick Vermersch

**Affiliations:** 1 Department of Neurology, EA2686, Université Lille Nord de France, Lille, France; 2 Department of Neuroradiology, Université Lille Nord de France, Lille, France; 3 French Reference Centre for Paraneoplastic Neurological Syndrome, Hospices Civils de Lyon, Hôpital Neurologique, F-69677, Bron, France; 4 Lyon Neuroscience Research Center INSERM U1028/CNRS UMR 5292, F-69372, Lyon, France; 5 Université de Lyon, Université Claude Bernard Lyon 1, F-69372, Lyon, France

**Keywords:** Myelitis, Anti-NMDAR antibodies, Encephalitis, Neuromyelitis optica

## Abstract

**Background:**

Encephalitis with anti-N-methyl-D-aspartate receptor antibodies (anti-NMDAR-Ab) is a rapid-onset encephalitis including psychosis, seizures, various movement disorders and autonomic system disturbances.

**Case presentation:**

We report a very unusual case of extensive myelitis associated with anti-NMDAR-Ab. MRI also revealed a hyperintense T2 lesion, non-suggestive of MS, which progressively extended, associated with periventricular gadolinium enhancement visualized on brain MRI. Ophthalmological evaluation showed subclinical right optic neuritis. The absence of anti-AQP4 antibody argued against neuromyelitis optica spectrum disorder. A slight psychomotor slowing prompted us to search for various causes of autoimmune encephalitis. Anti-NMDAR-Ab was found in cerebrospinal fluid.

**Conclusion:**

In patients with extensive myelitis who are seronegative for anti-AQP4 antibodies, and after other classical causes have been excluded, the hypothesis of atypical anti-NMDAR-Ab encephalitis should also be considered.

## Background

Encephalitis with anti-N-methyl-D-aspartate receptor antibodies (anti-NMDAR-Ab) is a rapid-onset encephalitis including psychosis, seizures, various movement disorders and autonomic system disturbances. The physiopathology is based on immune-mediated neuronal dysfunction [1,2]. We report the case of a 65-year-old woman presenting an extensive myelitis associated with anti-NMDAR-Ab.

## Case presentation

A 65-year-old woman was admitted because of progressive paraparesis (June 2012). Six months before admission, she had presented with fever, chills, abdominal pain and unusual headache for one week with spontaneous recovery. Abdomen and brain MRI were normal at that time. Two months before admission, she described a progressive onset of walking difficulties. At the same time, constipation and dysuria were noted. At her admission, neurological examination noted walking difficulties related to moderate paraparesis, moderate superficial and deep sensory dysfunction of the lower limbs and urinary retention. A very slight dysfunction of mental processing without speech disturbance was noted and confirmed by her family. No seizures, dyskinesia, movement disorders, psychiatric symptoms or autonomic dysfunction were observed.

**Figure 1:**
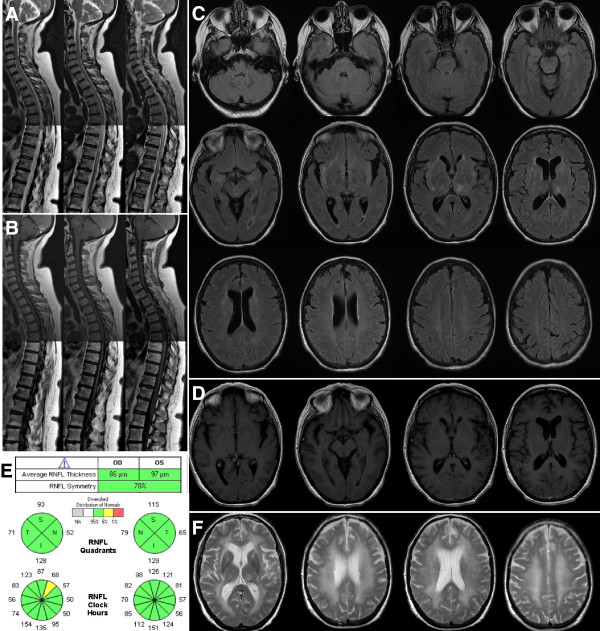
**Spinal cord, brain and optical imaging follow-up of the patient.** June 2012: Spinal cord magnetic resonance imaging (MRI) showing extensive hyper intense T2 lesion between C5 and T10 **(A)** with gadolinium enhancement on T1 sequence **(B)**. Brain MRI showing multiple hyperintense T2 lesions on insular regions, medial temporal lobes and thalamus **(C)**, but also gadolinium enhancement of ventricles and meninges **(D)**. Peripapillary optical coherence tomography showing asymmetric retinal nerve fiber layer **(E)**. July 2012: Control brain MRI showing extension of hyperT2 lesion load **(F)**.

Spinal cord MRI showed longitudinally extensive myelitis from C5 to T10 with gadolinium (Gd) enhancement (Figure 1A,B). Brain MRI follow-up showed T2 hyperintensities within the insular regions, medial temporal lobes and thalamus (Figure 1C), associated with gadolinium enhancement of the meninges and ventricles (Figure 1D). Analysis of the cerebrospinal fluid (CSF) showed moderate lymphocytic pleocytosis (53 cells/mm^3^), mild increased protein concentration (0.91 g/L) and oligoclonal bands. PCR assays for herpesviridae in CSF were all negative. Ophthalmological examination showed delayed P100 latencies on the right side on visual evoked potentials and asymmetric global retinal nerve fibre layer (RNFL) thicknesses on optical coherence tomography (OCT) suggestive of asymptomatic right optic neuritis (ON). OCT analyses were both perfectly centred on the optic nerve head (Figure 1E). Tests for anti-aquaporin 4 (AQP4) antibodies (Ab) in serum were negative. EEG showed slow and discontinuous activity in the left fronto-temporal regions. Extensive tests for auto-Ab (anti-onconeuronal, anti-DNA, anti-phospholipid, anti-voltage-gated potassium channel, anti-glutamate acid decarboxylase) were negative except for anti-NMDAR-Ab (IgG isotype) in the CSF and serum. No cancer was found (gynaecological examination, pelvic ultrasonography and MRI, mammography, total-body computed tomography scan and positron emission tomography scan were all normal).

Intravenous corticosteroids (1 g per day during 3 days plus 5 days) were given but the paraparesis worsened (bedridden patient). Brain and spinal cord MRIs follow-up (July 2012) showed an extension of T2 lesions without Gd enhancement (Figure 1F) and a stable extensive T2 lesion without Gd enhancement, respectively. As soon as anti-NMDAR-Ab were detected, plasma exchanges (PLEX) were initiated and followed by intravenous rituximab (375 mg/m^2^ every week for 4 weeks) plus tapering oral corticosteroids (1 mg/kg). Brain and spinal cord MRIs follow-up (August 2012) showed the disappearance of the extensive spinal cord T2 lesion and stable cerebral T2 lesions. Biological follow-up of anti-NMDAR-Ab rates in CSF and serum was performed (Table 1). CSF analysis was weakly positive just after corticosteroids/PLEX (M1) and was negative 2 months after rituximab (M3). Serum analysis was less correlated to clinical status. Four months after the inaugural myelitis, there was a significant improvement of the paraparesis (the patient was able to walk for 5 metres with bilateral support) and a total regression of the slight psychomotor retardation was observed. Unfortunately, the patient presented rapidly evolving pneumocystis pneumonia at this time and she died of respiratory failure. Autopsy was not performed.

**Table 1:** Anti-NMDA-R follow-up of the titration in CSF and in serum (qualitative and quantitative)

**Date**	**Jun 23rd**	**Jul 2nd**	**Jul 10th**	**Jul 11th**	**Jul 14th**	**Jul 16th**	**Jul 17th**	**Jul 23rd**	**Jul 30th**	**Aug 6th**	**Aug 13th**	**Oct 17th**
CSF	+ (1/10)	+ (low)	NA	NA	NA	NA	-	NA	NA	NA	NA	-
Anti-NMDA-R in serum	NA	NA	+ (1/10)	+ (1/10)	-	+ (low)	-	-	+ (low)	NA	+ (1/10)	-
Methylprednisolone (1 g/day IV)	3 days		5 days									
PLEX			Yes	Yes	Yes	Yes	Yes					
Rituximab (375 mg/m2/week during 4 weeks)								Yes	Yes	Yes	Yes	

Jun, June; Jul, July; Aug, August; Oct, October; CSF, cerebrospinal fluid; NA, not available; PLEX, plasma exchange; 5 PLEX were performed followed by infusions of rituximab (375 mg/m2/week during 4 weeks).

## Discussion

Our patient presented extensive myelitis associated with anti-NMDAR-Ab leading us to discuss a possible atypical form of anti-NMDAR-Ab encephalitis. Spinal cord symptoms were prominent throughout the clinical stage and were consistent with the extensive myelitis detected on spinal cord MRI. Slight psychomotor retardation is not sufficient to affirm that our patient presented anti-NMDAR-Ab encephalitis but early treatment might have stopped its development. Brain MRI showed hyperintense T2 lesions but also ventriculitis aspect. In view of the combination of extensive myelitis, brain T2 lesions non-suggestive of MS, ventriculitis aspect on MRI and subclinical ON, our first hypothesis was myelitis as a first event of neuromyelitis optica (NMO) or NMO spectrum disorder (NMOSD). Although cognitive disturbances are described in NMO [3], we also looked for other conditions involving auto-immune encephalitis and found anti-NMDAR-Ab in the CSF before immunosuppressive drugs had been applied.

Prodromal symptoms such as headache and fever are common [1] but we noted 3 atypical events for the hypothesis of anti-NMDAR-Ab encephalitis. First, myelitis is a very rare manifestation during anti-NMDAR-Ab encephalitis but has already been described [4-6]. In these 3 previous cases, all patients presented severe psychiatric or behavioural symptoms before the spinal cord syndrome and the encephalopathic symptoms remained prominent during all stages of the disease. In our case, the spinal cord syndrome remained prominent from the beginning and extension of the hyperintense T2 lesion was greater. Secondly, we highlighted in our case ventriculitis associated with meningeal Gd enhancement. Meningeal contrast enhancement in anti-NMDAR-Ab encephalitis is rare and [7,8], to the best of our knowledge, the ventriculitis aspect has never previously been described. NMDA receptors are membrane receptors widely expressed in the central nervous system on various cells such as neurons, oligodendrocytes but also astrocytes [9]. The ependymal regions contain ependymal cells and astrocytes rich in AQP4, and can also be involved in NMO, as described in 2 previously reported cases with thin ependymal and periventricular Gd enhancement [10,11]. Thirdly, our patient presented right subclinical ON. Since the prodromal symptoms dated back 6 months, it seems likely that the demyelinating pathology had already started 6 months before the spinal cord syndrome. RNFL atrophy most often (>94 %) appears within the 6 months after ON [12]. ON has only once been reported as a neurological manifestation of anti-NMDAR-Ab encephalitis [13].

Even though some correlations have been found between clinical status and anti-NMDAR-Ab titres in CSF, there is no reported correlation with anti-NMDAR-Ab titres in serum, and we therefore do not recommend a follow-up of anti-NMDAR-Ab titres in serum [1,14].

Anti-NMDAR-Ab encephalitis has been described following massive brain damage induced by herpes simplex encephalitis (HSE) [15,16]. Anti-NMDAR-Ab has also been found in sera of systemic lupus erythematosus and might be predictive of neuropsychiatric manifestations [17]. In the present case report, we cannot affirm that our patient presented anti-NMDAR-Ab encephalitis because of encephalopathic symptoms lacking and also cannot exclude that a massive brain and spinal damage of unknown etiology has led to secondary immunological response with anti-NMDA-R Ab production.

We can also discuss the hypothesis that our patient may have presented 2 auto-immune diseases concurrently: an NMOSD and an anti-NMDAR-Ab encephalitis. These are both Ab-mediated diseases and a comorbid association of auto-immune diseases is possible. Recently a case of NMO (seropositive anti-AQP4 Ab) following anti-NMDAR-Ab encephalitis has been described [18]. As our patient was seronegative for anti-AQP4 Ab and presented only subclinical ON, we cannot conclusively confirm or rule out this hypothesis.

## Conclusion

In patients with extensive myelitis who are seronegative for anti-AQP4 Ab, and after other classical causes have been excluded, the hypothesis of atypical anti-NMDAR-Ab encephalitis should also be considered.

## Consent

Written informed consent was obtained from the husband of the patient for publication of this Case report and the accompanying images. A copy of the written consent is available for review by the editor of this journal.

## Competing interests

The authors declare that they have no competing interests.

## Authors’ contributions

OO drafted the manuscript, participated to acquisition, analysis and interpretation of the data. GB participated to drafting and acquisition of data. JHod participated to acquisition of data and revised the manuscript for important intellectual concept. MG participated to acquisition of data and revised the manuscript for important intellectual concept. AL participated to acquisition of data and revised the manuscript for important intellectual concept. JHon revised the manuscript for important intellectual concept. HZ participated to analysis and interpretation of data and revised the manuscript for important intellectual concept. PV participated to acquisition of data and revised the manuscript for important intellectual concept. All authors read and approved the manuscript.

## Pre-publication history

The pre-publication history for this paper can be accessed here:

http://www.biomedcentral.com/1471-2377/13/211/prepub
